# COVID-19-Related Mortality Risk in People With Severe Mental Illness: A Systematic and Critical Review

**DOI:** 10.3389/fpsyt.2021.798554

**Published:** 2022-01-13

**Authors:** Marc De Hert, Victor Mazereel, Marc Stroobants, Livia De Picker, Kristof Van Assche, Johan Detraux

**Affiliations:** ^1^Department of Neurosciences, Center for Clinical Psychiatry, University Psychiatric Center, KU Leuven, Kortenberg, Belgium; ^2^Antwerp Health Law and Ethics Chair, University of Antwerp, Antwerp, Belgium; ^3^Biomedical Library, University Psychiatric Center, KU Leuven, Kortenberg, Belgium; ^4^Collaborative Antwerp Psychiatric Research Institute, University of Antwerp, Antwerp, Belgium; ^5^University Psychiatric Hospital Campus Duffel, Duffel, Belgium; ^6^Research Group Personal Rights and Property Rights, Faculty of Law, University of Antwerp, Antwerp, Belgium; ^7^Department of Neurosciences, Public Health Psychiatry, University Psychiatric Center, KU Leuven, Kortenberg, Belgium

**Keywords:** severe mental illness, schizophrenia, bipolar disorder, major depressive disorder, mortality, COVID-19

## Abstract

**Background:** Increasing clinical evidence suggests that people with severe mental illness (SMI), including schizophrenia spectrum disorders, bipolar disorder (BD), and major depressive disorder (MDD), are at higher risk of dying from COVID-19. Several systematic reviews examining the association between psychiatric disorders and COVID-19-related mortality have recently been published. Although these reviews have been conducted thoroughly, certain methodological limitations may hinder the accuracy of their research findings.

**Methods:** A systematic literature search, using the PubMed, Embase, Web of Science, and Scopus databases (from inception to July 23, 2021), was conducted for observational studies assessing the risk of death associated with COVID-19 infection in adult patients with pre-existing schizophrenia spectrum disorders, BD, or MDD. Methodological quality of the included studies was assessed using the Newcastle-Ottawa Scale (NOS).

**Results:** Of 1,446 records screened, 13 articles investigating the rates of death in patients with pre-existing SMI were included in this systematic review. Quality assessment scores of the included studies ranged from moderate to high. Most results seem to indicate that patients with SMI, particularly patients with schizophrenia spectrum disorders, are at significantly higher risk of COVID-19-related mortality, as compared to patients without SMI. However, the extent of the variation in COVID-19-related mortality rates between studies including people with schizophrenia spectrum disorders was large because of a low level of precision of the estimated mortality outcome(s) in certain studies. Most studies on MDD and BD did not include specific information on the mood state or disease severity of patients. Due to a lack of data, it remains unknown to what extent patients with BD are at increased risk of COVID-19-related mortality. A variety of factors are likely to contribute to the increased mortality risk of COVID-19 in these patients. These include male sex, older age, somatic comorbidities (particularly cardiovascular diseases), as well as disease-specific characteristics.

**Conclusion:** Methodological limitations hamper the accuracy of COVID-19-related mortality estimates for the main categories of SMIs. Nevertheless, evidence suggests that SMI is associated with excess COVID-19 mortality. Policy makers therefore must consider these vulnerable individuals as a high-risk group that should be given particular attention. This means that targeted interventions to maximize vaccination uptake among these patients are required to address the higher burden of COVID-19 infection in this already disadvantaged group.

## Background

People with severe mental illness (SMI), including schizophrenia (SZ) (lifetime prevalence: 0.7%), bipolar disorder (BD) (lifetime prevalence: 0.4–1.1%), and major depressive disorder (MDD) (lifetime prevalence: 15–18%) (1–3), have a two to three times higher mortality rate than the general population (4–7). This mortality gap translates into a 10–20 years shortened life expectancy (6, 8) and appears to be widening (9).

It is well-known that the majority of deaths in individuals with SMI are due to physical diseases, predominantly cardiovascular diseases (8, 10). Non-medical factors, including unhealthy lifestyles, disparities in physical health care, and stigmatizing attitudes toward people with SMI, contribute to the higher risk of death (11–13). Disease-related factors, such as unawareness of physical problems and challenges in appraising health information due to cognitive deficits, delusions, and, in general, lower educational attainment and health literacy (11, 14–18), as well as the use of psychotropic medication (antipsychotics, antidepressants, and mood stabilizers) (4, 10, 12) may further increase the risk of physical comorbidities.

Research has shown that several respiratory viruses, including human coronaviruses, can have neuroinvasive properties (19). The central nervous system (CNS) is also a potential target for the SARS-CoV-2 virus, because angiotensin-converting enzyme 2 (ACE 2) receptors, used by the virus to enter the cells, are equally expressed in glial cells and neurons in the brain (20–23). Although there is still no convincing evidence for direct neuropathogenic effects of SARS-CoV-2 (24, 25), COVID-19 infection can cause CNS damage (26–28). In a prospective autopsy cohort study, extensive inflammatory changes, affecting both white and gray matter, were detected in the brain of patients with lethal COVID-19. This inflammatory response was most pronounced in the olfactory bulbs and medulla oblongata. Results of this study suggest that CNS changes are due to a maladaptive immune response, rather than the consequence of a direct virus-induced effect, given that viral presence was low at late stages of COVID-19 (29). The first longitudinal imaging study (which had not been peer reviewed as of Oct 13, 2021), comparing structural and functional brain scans acquired from individuals before and after SARS-CoV-2 infection (*n* = 401) with scans from a well-matched control group (*n* = 384), demonstrated brain atrophy, mainly in the limbic regions with direct neuronal connectivity to the primary olfactory and gustatory system, in addition to a more diffuse loss of gray matter. These authors suggested that the observed brain changes may be due to a direct virus effect, or to neuroinflammation, following viral infection and initiating chronic neuronal dysfunctions (30).

Different pathogenic pathways may be involved (31). One of the proposed mechanisms is that the SARS-CoV-2 virus enters the CNS through the neuronal retrograde route. In this case the virus infects neurons in the periphery and uses the olfactory nerve pathway to gain access to the CNS and cause infections of immune-functioning microglia or astrocytes in the CNS (19, 30). This, however, does not rule out a pathway from the nose to the brain by other mechanisms (such as the vascular route). A review by Uversky et al. (20) stated that there are at least seven candidate routes the SARS-CoV-2 virus can use to reach the CNS.

Nevertheless, because of these findings concerns have been raised regarding the neuroinvasive effect of COVID-19 infection in patients with pre-existing neuropsychiatric disorders, in particular in SMIs, which are often already characterized by a systemic pro-inflammatory state (32, 33). According to one review, 0.9–4% of individuals infected with SARS-CoV-2 develop psychotic spectrum disorders (34). A retrospective cohort study reported the following numbers (<6 months after acute infection): 0.9% in COVID-19 infection without hospitalization, 2.9% after hospitalization, and 7% after COVID-19-related encephalopathy (28). However, some of these cases probably are due to COVID-19-related psychosocial stress or treatment (e.g., steroid treatment in patients with COVID-19) (34, 35).

Recently, five systematic reviews and meta-analyses (36–40), assessing the risk of COVID-19-related mortality in patients with a psychiatric disorder, have demonstrated that people with SMI have a higher COVID-19-related mortality risk, compared to general population controls, and even compared to people with other psychiatric disorders. This risk remained high after adjustment for age, sex, and other confounders. Although these reviews have been thoroughly conducted, certain methodological limitations may hinder the accuracy of their research findings. Most of these reviews did not use a comprehensive search strategy for COVID-19 (36–39), or did not include the Embase database (36, 37, 40). Several of these reviews not only included laboratory-confirmed COVID-19 cases, but also patients where the SARS-CoV-2 infection was based on a clinical diagnosis made by physicians (36, 37, 39), or did not make this clear in their methodology section (40). Furthermore, reviews reporting mortality data on mood disorders did not distinguish unipolar and BDs (although most of the included studies in these reviews relied on electronic medical records that do not allow a fine-grained analysis of clinical variables) and/or pre-existing from post-infection or comorbid disorders [e.g., (38)]. Finally, a thorough discussion part is also missing in most of these reviews.

## Aim of The Study

To overcome the above-mentioned limitations a novel systematic literature search was conducted to assess the risk of death associated with COVID-19 infection in people with SMI (schizophrenia spectrum disorders, BD, and MDD), compared with patients without SMI, or without any psychiatric disorder, and a thorough discussion part was provided.

## Methods

### Search Strategy

A comprehensive literature search, using the PubMed, Embase, Web of Science, and Scopus databases (from inception to July 23, 2021), was conducted without language restriction for studies reporting data on the risk of death associated with COVID-19 infection in adult patients with schizophrenia spectrum disorders, BD, or MDD, compared with controls (patients without a SMI or without a psychiatric disorder). Two of the authors (JD and MD) and two experienced biomedical information specialists worked closely together to construct effective search strings for the different databases. Full search strategies are available as Supplementary Material 1. Duplicates were removed using EndNote X9 and Rayyan QCRI (JD). After removing duplicates, titles and abstracts were screened by JD. Articles that were deemed potentially relevant according to the selection criteria were selected. JD and MD independently reviewed the full text of the selected articles and assessed their eligibility. They also attempted to identify additional studies through a systematic search of the reference lists of selected articles and of previously published systematic reviews/meta-analyses.

### Selection Criteria

Inclusion criteria were:

- Published, peer-reviewed, original studies,
- Population-based observational studies, including case-control, cohort, or cross-sectional studies,
- Studies including patients with laboratory-confirmed COVID-19 cases (i.e., a positive real-time reverse transcription-polymerase chain reaction test),
- Studies including patients with a clinically confirmed pre-existing SMI (i.e., schizophrenia spectrum disorders, BDs, and/or unipolar depression), using a widely-accepted standardized disease coding system, such as the Diagnostic and Statistical Manual of Mental Disorders (DSM) or the International Classification of Diseases (ICD), and
- Studies reporting COVID-19-related mortality outcomes [i.e., odds ratio, risk ratio, hazard ratio (HR), or associated metrics] and comparing COVID-19-related mortality risks of SMI patients with non-SMI patients, or patients without a psychiatric disorder.

Studies that did not include patients with pre-existing SMI (thus studies where COVID-19 may have been an antecedent to the onset of the SMI disorder), a control group, COVID-19-related mortality outcomes, original data, or were not peer-reviewed and published (preprints, conference papers), as well as case reports, reviews, meta-analyses, and studies where SMI mortality outcomes were grouped with those for non-SMIs, or studies where COVID-19 was not ascertained according to laboratory testing were excluded.

This systematic review adhered to the 2020 Preferred Reporting Items for Systematic Review and Meta-Analyses (PRISMA) guidelines (41). We did, however, not register our protocol prior to submitting the manuscript for publication.

### Data Extraction

Data from the included studies were extracted from each article by JD to collect the following information:

- Study characteristics: author information, publication year, country where the study was conducted, study design (cross-sectional, cohort or case-control, prospective vs. retrospective), time period studied during the COVID-19 pandemic,
- Patient characteristics: primary diagnoses included in the study, sample sizes of SMI patients and controls, mean/median age (years), and sex distribution,
- Outcome measure: adjusted and unadjusted mortality data (odds ratio, risk ratio, HR, or associated metrics with 95% confidence interval estimates), and
- Covariates in adjusted risk.

### Assessment of Risk of Bias

The quality of each observational study was rated using the Newcastle-Ottawa Scale (NOS), whereby a higher score indicated higher methodological quality and lower risk of bias. The NOS was adapted for cross-sectional data.

## Results

### Search Strategy

**Figure 1:**
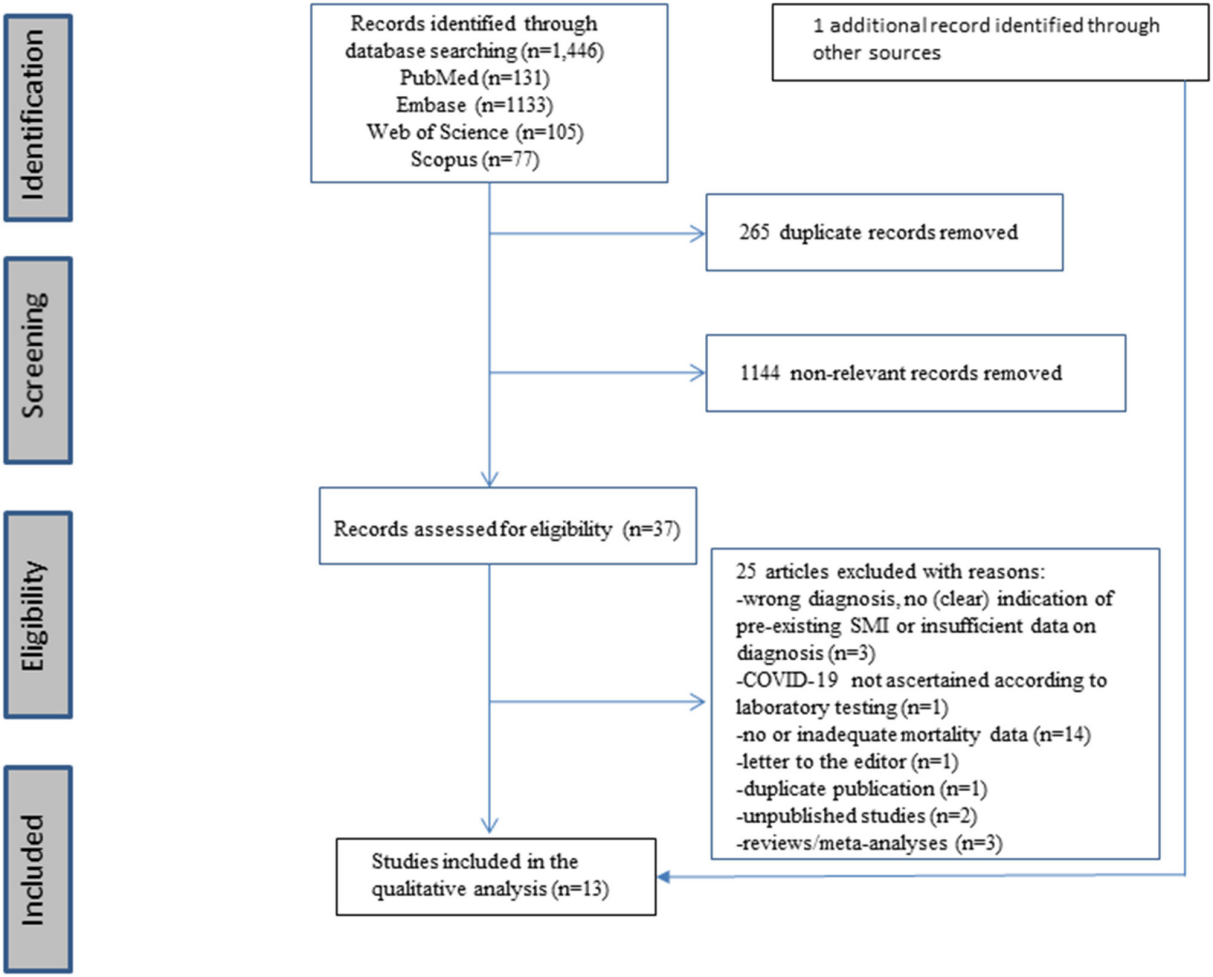
Prisma checklist flow diagram.

The original search in the PubMed (*n* = 131), Embase (*n* = 1,133), Web of Science (*n* = 105), and Scopus (*n* = 77) databases yielded a total of 1,446 reports. Of these, 265 duplicate reports were removed. Overall, 37 references of published studies were selected as potentially eligible, of which 12 original records met the inclusion criteria. One record, identified through cross-referencing and which fulfilled the inclusion criteria, was added, resulting in a total of 13 original records. The results of the study selection are shown in the PRISMA flow diagram (see Figure 1).

### Study and Patient Characteristics

Study and patient characteristics, as well as mortality data and covariates are presented in Table 1. Median age of SMI patients across studies ranged from 40 to 66 years and was not reported in nine studies. Two studies were carried out in Denmark, two in France, one in Israel, one in South Korea, two in Spain, one in the U.K., and four in the U.S. Severe mental illness was almost exclusively categorized according to ICD classification criteria. In the French and Israeli studies only patients with schizophrenia (spectrum disorders) were analyzed. The U.S. study of Egede et al. (46) analyzed exclusively data of BD patients.

**Table 1:** Study characteristics and mortality data of the included studies (33, 42–53).

**Study**	**Country**	**Study design**	**Time period**	**Diagnosis**	**Sample size**	**Mean/median age (years) Male gender**	**Risk (95% CI)**	**Covariates in adjusted risk**
Barcella et al. (42)	Denmark	Cohort study	February 27 2020–January 2 2021	Schizophrenia spectrum disorders (F20–29) BD (F30, 31, 38) Unipolar depression, including MDD (F32–34, F39) (ICD-8 and ICD-10) Controls = patients without psychiatric disorder	Schizophrenia spectrum disorders (*n* = 984) vs. controls (*n* = 127,281) BD (*n* = 485) vs. controls (*n* = 127,281) Unipolar depression (*n* = 3,764) vs. controls (*n* = 127,281)	40 (median) 48.8% 45.3 (median) 37.3% 44 (median) 30.8%	**Standardized average RR: 2.29 (1.36–3.22)** **Unadjusted HR:4.12 (2.64–6.43)** **Adjusted HR:2.41 (1.53–3.79)** **Standardized average RR: 1.87 (1.12–3.12)** **Unadjusted HR:3.34 (1.66–6.70)** Adjusted HR:1.94 (0.97–3.90) **Standardized average RR: 1.92 (1.39–2.44)** **Unadjusted HR:2.63 (1.97–3.52)** **Adjusted HR:2.04 (1.52–2.74)**	Age, sex, highest obtained education, income, ischemic heart disease, congestive heart failure, cerebrovascular disease, CKD, HP, peripheral artery disease, DM, COPD, asthma, substance abuse, and malignancy
Nemani et al. (43)^†^	U.S.	Retrospective cohort study	March 3 2020–May 31 2020	Schizophrenia spectrum disorders (F20, 22, 23, 25, 28, 29) Mood disorders -Unipolar depression, including MDD F32, 33, 34, 39 -BD F30, 31 (ICD-10) Controls = patients without psychiatric disorders, with the exception of patients with organic mental disorders, mental disorders due to substance use, mental retardation, and disorders of psychological development.	Lifetime SZ vs. controls: 75/6,349 Lifetime mood disorders vs. controls: 564/6,349 Recent SZ vs. controls: 46/6,349 Recent mood disorders vs. controls: 374/6,349	N/A	**Unadjusted OR: 2.93 (1.75–4.92)** **Demographically adjusted OR: 2.87 (1.62–5.08)** **Fully adjusted OR: 2.67 (1.48–4.80)** **Unadjusted OR: 1.82 (1.45–2.29)** Demographically adjusted OR: 1.25 (0.98–1.61) Fully adjusted OR: 1.14 (0.87–1.49) **Unadjusted OR: 2.84 (1.47–5.52)** **Demographically adjusted OR: 3.13 (1.50–6.54)** **Fully adjusted OR: 2.67 (1.26–5.69)** **Unadjusted OR: 2.19 (1.69–2.84)** **Demographically adjusted OR: 1.52 (1.13–2.03)** Fully adjusted OR: 1.27 (0.94–1.73)	Demographically adjusted: age, sex, race Fully adjusted (demographically + medical risk factors): age, sex, race, smoking status, HP, heart failure, myocardial infarction, DM, CKD, COPD, and cancer
Tzur Bitan et al. (44)	Israel	Retrospective cohort study	March 2020–October 2020	SZ (ICD-9 or ICD-10) (F20) Controls = people without schizophrenia randomly drawn from the general population	642 patients vs. 709 controls	51.51 (mean, SZ) 51.37 (mean, controls) 61%	**Non-adjusted OR: 3.14 (1.34–7.36)** **Adjusted OR (95% CI): 3.27 (1.39–7.68)**	Age, sex
Jeon et al. (45)	South Korea	Retrospective cohort study	1 December 2019–15 May 2020	Schizophrenia spectrum disorders (ICD-10) (F20–F29) Mood disorders (BD+ MDD) (ICD-10) (F30-F39) Controls = patients without a psychiatric disorder	159 patients vs. 628 controls 273 patients vs. 1,060 controls	N/A	Adjusted OR: 2.25 (0.36–14.03) Adjusted OR (95% CI): 2.33 (0.96–5.66)	Cohort matched by age, sex, and Charlson Comorbidity Index with up to four people without mental disorder and adjusted for type of insurance, medical history of DM and pneumonia, and use of β-blockers and anticonvulsants
Egede et al. (46)	U.S.	Cross-sectional analysis	March 20–July 10 2020	BD (ICD-9 and ICD-10) (F30.1–F30.4, F30.9, F31.1–F31.6, F31.73–F31.78, F31.9) Controls = patients without a psychiatric disorder	38 patients vs. 1,330 controls	52.3 (mean) 29.7%	**Non-adjusted HR: 2.83 (1.15–6.96)** **Demographically adjusted HR: 2.63 (1.07–6.49)** **Fully adjusted HR: 2.67 (1.07–6.67)**	Gender, age, race/ethnicity, location, and primary payor, tobacco use, and BMI.
Fond et al. (47)	France	Case-control study	February 27 2020–May 4 2020	SZ (F20,22,25) (ICD-10) Controls = patients without a psychiatric disorder	15 patients vs. 1,077 controls	66 (median) 73.3%	**Non-adjusted OR: 3.80 (1.19–12.20)**	
							**Adjusted OR: 4.36 (1.09–17.44)**	Age, sex, smoking status, obesity, Charlson Comorbidity Index
							**Adjusted OR: 4.28 (1.07–17.20)**	Age, sex, smoking status, obesity, Charlson Comorbidity Index, hydroxychloroquine
							**Adjusted OR: 4.33 (1.08–17.34)**	Age, sex, smoking status, obesity, Charlson Comorbidity Index, hydroxychloroquine-azithromycin combination
Fond et al. (48)	France	Cohort study	February 1 2020–June 9 2020	SZ (F20,22,25) (ICD-10) Controls = patients without a SMI	823 patients vs. 49,927 controls	48.8%	**Unadjusted OR: 1.25 (1.05–1.49)** **Adjusted OR: 1.30 (1.08–1.56)** Significant interaction between SZ and age (*p* = 0.0006): SZ patients between 65 and 80 years have a significantly higher risk of death than controls of same age [**+7.89%; Adj. OR: 1.62 (1.28–2.06)]**	Age, sex, social deprivation, smoking status, overweight and obesity, Charlson Comorbidity Index, origin of the patient, hospital category, number of hospital stays for COVID-19, geographical areas of hospitalization
Reilev et al. (49)	Denmark	Cohort study	February 27 2020–May 19 2020	SMI=SZ (F20), schizoaffective disorder (F25), or BD (F30,31)(ICD-10) Controls = patients without a SMI	76 patients vs. 11,046 controls	N/A	**Non-adjusted OR: 3.8 (2.1–7.0)** **Demographically adjusted OR: 2.5 (1.2–5.1)** Fully adjusted OR: 1.9 (0.9–3.9)	
								Age, sex
								Age, sex, and number of comorbidities
Poblador-Plou et al. (50)	Spain	Retrospective cohort study	March 4 2020–May 17 2020	Mood disorders (ICD-9-CM) Controls = patients without a psychiatric disorder	202 patients vs. 569 controls	N/A	Adjusted OR: 1.38 (0.98–1.95) (men with mood disorder) **Adjusted OR: 1.46 (1.12–1.91) (women with mood disorder)**	Age Age
Yang et al. (33)	U.K.	Retrospective cohort study	January 31 2020–July 26 2020	MDD (ICD-9 or ICD-10) Psychotic disorders (ICD-10) Controls = non-psychiatric patients	22,352 patients vs. 398,662 controls 1,431 patients vs. 419,583 controls	N/A	**Adjusted OR: 2.68 (2.03–3.54)** **Adjusted OR: 3.50 (1.70–7.17)**	Adjusted for birth year, sex, race or ethnicity, Townsend deprivation index, educational attainment, annual household income, BMI, smoking status, and history of chronic cardiac disease, DM, COPD, CKD, and asthma.
Castro et al. (51)	U.S.	Retrospective cohort study	February 15 2020–May 24 2020	Mood disorders (MDD and BD) (ICD-10) Controls = individuals without a psychiatric disorder	717 patients vs. 2,271 controls	N/A	Mortality risk ≥hospital *day* 12 **Unadjusted HR: 2.16 (1.54–3.02)** **Demographically adjusted HR: 2.00 (1.42–2.82)** **Fully adjusted HR: 1.54 (1.05–2.25)** Mortality risk <hospital *day* 12 Unadjusted HR: 1.17 (0.91–1.50) Demographically adjusted HR: 1.08 (0.83–1.39) Fully adjusted HR: 0.88 (0.65–1.18)	Age, sex, race, ethnicity, admission site (academic medical center compared with community hospital), socioeconomic status, and Charlson comorbidity index
Diez-Quevedo et al. (52)	Spain	Retrospective cohort study	March 1 2020–November 17 2020	Mood disorders (ICD-10) Controls = patients without mood disorders	279 patients (mood disorder) vs. controls	N/A	**Adjusted HR: 1.52 (1.13–2.06)**	Sex, age, history of medical and psychiatric disorders
Wang et al. (53)	U.S.	Retrospective case-control study	Up to July 29 2020	Depressive disorder (code 3548900) (including MDD) (SNOMED-CT) Controls = patients without a psychiatric disorder	1,460 patients with a recent (past year, but prior to COVID-19) diagnosis of depression	N/A	**Unadjusted OR: 1.56 (1.28–1.91)**	Age, sex, ethnicity, and medical comorbidities (cancers, CVDs, type 2 DM, obesity, CKD, COPD, asthma, and SUDs)

*BD, bipolar disorder; BMI, body mass index; CI, confidence interval; CKD, chronic kidney disease; COPD, chronic obstructive pulmonary disease; CVD, cardiovascular diseases; DM, diabetes mellitus; HP, hypertension; HR, hazard ratio; ICD-9-CM, International Classification of Diseases, Ninth Revision, Clinical Modification; ICD, International Classification of Diseases; MDD, major depressive disorder; N/A, not available; OR, odds ratio; RR, risk ratio; SMI, severe mental illness; SNOMED-CT, systematized nomenclature of medicine-clinical terms; SUDs, substance use disorders. Bold values indicate statistical significance between groups*.

† *A secondary analysis was limited to patients with recently documented psychiatric diagnoses of interest recorded in an encounter between January 1, 2019, and March 3, 2020 (recent diagnoses)*.

### Quality Assessment

Data on study quality are presented in Supplementary Material 2. Methodological quality was high in nine studies, and moderate in four studies.

### Study Results

For studies analyzing mortality data separately for patients with schizophrenia spectrum disorders, BD and MDD, fully adjusted risks (= adjusted for demographic factors and one or more comorbidities or other covariates) ranged from 1.30 to 4.36 for schizophrenia spectrum disorders, and from 2.04 to 2.68 for depression. Only two studies (42, 46) reported COVID-19-related mortality data separately for patients with BD. In the study of Egede et al. (46), including exclusively BD patients, a HR of 2.67 (95% CI: 1.07–6.67) was found, while Barcella et al. (42) did not find a significant HR [adjusted HR = 1.94 (0.97–3.90)] in the fully adjusted model. A variety of factors were likely to contribute to the increased mortality risk of COVID-19 in these patients. These included male sex, older age, and somatic comorbidities, as evidenced by the reduction of the unadjusted risk after adjusting for these demographic factors and comorbidities. However, as even after this adjustment the risk of COVID-19-related mortality was still increased, other factors also seem to play a primordial role (see Table 1).

## Discussion

Our systematic review shows that, after full adjustment for relevant confounders, the extent of the variation in COVID-19-related mortality rates between studies including people with schizophrenia spectrum disorders was large. COVID-19-related mortality risk was found to be 2- to 4-fold increased for patients with schizophrenia spectrum disorders, compared with controls. There are several reasons for this variation:

1. studies showing higher adjusted mortality estimates included rather small samples of patients (47), and/or presented mortality data with large confidence intervals (33, 45, 47), indicating a low level of precision of the estimated mortality outcome(s),
2. while some studies were strictly limited to patients with SZ (44), others included disorders covering more or less the whole spectrum of SZ-like disorders (42, 43, 45). In addition, there is (besides the lack of information about psychopharmacological treatments and psychiatric treatment settings of patients) a lack of specific information about the severity and the status (first-episode vs. chronic) of the disease. These elements are important to consider since different forms of the disorder may have different risks of COVID-19-related mortality, and
3. the comparison group sometimes included non-SMI patients and at other times patients without a psychiatric disorder.

Although results are more stable for studies on MDD (COVID-19-related mortality risk in these patients seems to be 1.5- to 2-fold increased, compared with controls), these studies also in most cases did not include specific information on the mood state or disease severity of the patients. While some studies were strictly limited to patients with MDD (33, 51), others (42, 43) also included mild or moderate forms of the disease.

Finally, several studies (45, 49, 50, 52) involved a mixed population and did not make a distinction between the three main categories of SMIs. Studies on BD patients clearly are lacking.

Recently, several systematic reviews and meta-analyses (36, 37, 39, 40), showed that pre-existing mental disorders were associated with an increased COVID-19-related mortality risk, compared to controls, even after adjustment for age, sex, and other confounders. In the meta-analysis of Fond et al. (37), patients with SMI (schizophrenia spectrum disorders and/or BD) were found to have the highest risk of COVID-19-related mortality (adjusted OR = 1.67; 95% CI: 1.02–2.73). Vai et al. (36) also observed that the most robust associations were found for psychotic disorders (adjusted OR = 1.68; 95% CI: 1.29–2.18) and mood disorders (adjusted OR = 1.43; 95% CI: 1.15–1.79), after adjustment for age, sex, and other confounders, with a statistically significant difference (*p* = 0.0047) identified between adjusted estimates for SMI patients (adjusted OR = 1.55; 95% CI: 1.30–1.85) and patients with non-SMI psychiatric disorders (adjusted OR = 1.09; 95% CI: 0.92–1.29). A very recently published cohort study confirmed the previously published evidence (54). These results thus show that patients with SMI have a statistically significantly higher risk of death than patients with non-SMI disorders. Moreover, analyses stratified by the number of redeemed psychotropic medications indicated that COVID-19-related mortality risk increases with higher psychotropic medication use (42). All these results therefore suggest an association between mental illness severity and COVID-19-related mortality risk.

A retrospective cohort study in patients with mood disorders has found that COVID-19-related mortality risk in these patients seems to be particularly elevated 2 weeks after admission, while there seems to be little difference in mortality risk with controls during early hospitalization (51). However, the meta-analysis of Vai et al. (36) found no evidence of increased in-hospital mortality in patients with psychiatric disorders vs. those without. Moreover, COVID-19-related mortality risk was significantly higher among psychiatric patients who were not admitted to the hospital than among hospitalized patients.

### Factors Underlying the Association Between SMI and COVID-19-Related Mortality

Several demographic factors and somatic comorbidities have been identified that contribute to the higher observed mortality estimates associated with COVID-19 infection in people with SMI (42–44, 49, 50, 53, 55, 56) and without SMI (57–63). These include older age (≥65–70 years old), male gender, low socioeconomic status (SES) and educational level, and physical diseases (cardiovascular disease, hypertension, chronic obstructive pulmonary disease, chronic kidney disease, diabetes). These identified factors may have a more profound impact on people with a SMI.

Firstly, as in the general population, particularly older individuals (≥60 years) with SMI are vulnerable to COVID-19. However, there seems to be an excess mortality due to COVID-19 among patients of this age group. Deaths due to COVID-19 were found to be 4 times higher for those with SMI, compared to individuals without SMI within the same age group [people with SMI: 0.01% (40–59 years), 0.12% (60–69 years), 0.46% (70–79 years), vs. individuals without SMI: 0.01% (40–59 years), 0.03% (60–69 years), 0.11% (70–79 years)] (55). Fond et al. (48) found that patients with SZ between 65 and 80 years had a higher risk of death [7.69% (<55 years) and 30.29% (≥65 <80 years)], compared to individuals without a SMI of the same age [4.04% (<55 years) and 22.4% (≥65 <80 years)] [Adj. OR (95% CI): 1.62 (1.27–2.06, *p* = 0.0002)]. These results can be linked to the accelerated biological aging hypothesis, one of the major causes of the higher premature mortality rates that are observed in people with SMI (64, 65). This means that aging of both body and brain, and, in particular for COVID-19, the senescence of immune cells (66), might be more rapid in these people. One study found that molecular brain age (i.e., biological age of the brain) was 2–6 years higher than the chronological age in individuals with SZ, and 4.7–7.5 years higher in subjects with BD. No increase in brain aging was noted in subjects with MDD (67).

The impact of SES, which has been found to be associated with health care access (68), on the risk of COVID-19-related mortality remains to be elucidated, particularly in individuals with SMI (55). Due to the complexity of SES and its metrics (such as the Townsend Deprivation Index or the Distressed Communities Index), it stays unclear which individual components are associated with COVID-19-related mortality. While the impact of certain socioeconomic aspects (e.g., lower education and race) on COVID-19-related mortality has been shown (69, 70), other components (such as poverty and unemployment) were found to be protective against COVID-19-related mortality (69). Moreover, these measurements remain indirect indices of health care access and are assessed only once at baseline. Therefore, misclassification due to the absence of repeated measurements might exist (33). The same applies to the impact of the type of care/facility on the treatment outcomes for persons with SMI with COVID-19, particularly during the first wave of the COVID-19 pandemic. At the beginning of this pandemic, in the U.S. (71) and in Europe (72) many inpatient psychiatric facilities created psychiatric COVID-19-positive units (PCU). When tested positive, psychiatric patients were transferred to these units, where they were treated medically for their COVID-19 illness by internists and medical nurse practitioners. Only if patients showed signs of respiratory distress, such as shortness of breath or chest pain, they were transferred to a medical emergency room for further evaluation (71). The organization of PCU, however, varied considerably across countries and over time (72). Therefore, it remains unknown what the effect is of the type of care/facility on COVID-19-related mortality figures in these persons.

Secondly, a higher somatic comorbidity burden in patients with SMI, compared to non-psychiatric patients, may also partly explain the increased COVID-19-related mortality risk. However, with the exception of cardiovascular diseases, results for other somatic comorbidities are sometimes inconsistent. In SMI and non-psychiatric populations hypertension, diabetes, and chronic obstructive pulmonary disease have been found to correlate with an increased risk of fatality in most (43, 49, 73–75), but not all studies (55, 76). Nevertheless, it is well-recognized that individuals with these comorbidities are at an increased risk for a severe course of COVID-19 (77). In addition, somatic comorbidities seem in general to be an important driver of the observed increased COVID-19-related infection and mortality estimates in patients with SMI (33, 43, 49, 53). The reasons why certain somatic comorbidities are associated with more severe COVID-19 illness in people with SMI are not yet fully understood (78).

Although results are inconsistent, the use of psychotropic medications may be another important risk factor. A recent meta-analysis (36) showed that, after adjustment for age, sex, and other confounders, COVID-19-related mortality was associated with exposure to antipsychotics (initiated before contracting COVID-19) (adjusted OR = 2.43, 95% CI: 1.81–3.25), but not to antidepressants (adjusted OR = 1.18, 95% CI: 0.93–1.50). However, a very recently published retrospective cohort study did not observe an association between antipsychotic use and COVID-19-related mortality (79). An important obstacle in finding an answer to the question whether there exists an association between the use of psychotropic medications and COVID-19-related mortality is that specific data on the use of psychotropic medication and psychiatric status (acute phase vs. stabilization phase) across studies is lacking.

Some antipsychotic medications (particularly clozapine) seem to increase susceptibility to pneumonia and pneumonia-related mortality risk in individuals with SMI due to sedation, impaired swallowing and hypersalivation (80–82). Clozapine can also suppress immune function (78). In animal models, short-to intermediate-term exposure to clinically relevant levels of risperidone has been shown to induce inflammatory and adaptive immune process dysregulation, possibly affecting susceptibility to respiratory infections, including COVID-19 (83). Nevertheless, some studies found a protective effect for COVID-19 infection in patients treated with antipsychotics (84, 85). Based on preclinical findings, the antipsychotics chlorpromazine and haloperidol have been suggested to offer protection against SARS-CoV-2, possibly through their interactions with sigma-1 receptors, inducing anti-inflammatory effects by inhibiting cytokine production (86–90). However, initial observational clinical studies did not confirm that these agents offer protection against COVID-19 infection or COVID-19-related mortality (84, 91). However, the results of these studies have to be interpreted with caution, because of possible confounding factors. Moreover, it is important to make a distinction between acute and long-term treatment effects of antipsychotic treatment.

Valproate, a mood stabilizer, also may be associated with an increased risk of respiratory infections (92). By contrast, lithium, another mood stabilizer, seems to be associated with a decreased risk of respiratory infections and demonstrated potential antiviral properties at a preclinical level (92, 93). Lithium has even been proposed as a candidate treatment for COVID-19. It can suppress NOD-like receptor family pyrin domain containing-3 (NLRP3) inflammasome activity (which is implicated in the release of pro-inflammatory cytokines during the cytokine storm), inhibits cell death (resulting in a decrease in lung parenchymal damage), and is characterized by immune-regulatory mechanisms (preventing the harmful effects of immune hyperactivation) (94). However, its antiviral properties, as well as its safety as a potential antiviral agent (due to its narrow therapeutic index and high risk of toxicity), remain to be confirmed in clinical settings (93, 95).

More promising is the association that has been observed between FIASMA (Functional Inhibitors of Acid SphingoMyelinAse) treatments, including certain SSRI and non-SSRI antidepressants such as fluvoxamine and amitriptyline, and a reduction in clinical deterioration and mortality risk in patients with COVID-19. Acid sphingomyelinase (ASM) is an important lipid-metabolizing enzyme catalyzing the hydrolysis of sphingomyelin into ceramide and phosphorylcholine (91, 96). SARS-CoV-2 probably activates this ASM-ceramide system, facilitating viral entry and infection of human nasal epithelial cells by clustering ACE-2 receptors (97, 98). Functional Inhibitors of Acid SphingoMyelinAse antidepressants are thought to impair SARS-CoV-2 entry into epithelial cells by functional inhibition of the ASM-ceramide system (99). Several retrospective (100–102) and prospective (103) observational studies, as well as a small double-blind randomized trial (104) showed that taking a FIASMA treatment was associated with a lower risk of clinical deterioration or death in both non-psychiatric and psychiatric patients with COVID-19. The anti-inflammatory properties of certain antidepressants, probably due to their high affinity for sigma-1 receptors (105), may have additional value in managing COVID-19. Nevertheless, more large-scale double-blind controlled randomized clinical trials of these medications in patients with COVID-19 are needed (99, 106, 107).

Polypharmacy has been found to be associated with a higher risk of developing COVID-19 (108). Psychotropic polypharmacy is quite common in patients with SMI (12). According to a Swedish study, 25% of patients dispensed antipsychotic drugs receive a combination of two or more antipsychotic drugs. These patients also did more often take anxiolytics and sedatives than those prescribed antipsychotic monotherapy (109). Psychotropic polypharmacy, particularly during the treatment of elderly people with SMI, seems to be associated with greater adverse effects on most physical diseases, compared to monotherapy (4), as it carries the risk of adverse drug reactions. As the possible contribution of antipsychotic polypharmacy to the general excess mortality in people with SMI remains unclear (110, 111), further meta-analyses are needed analyzing mortality outcomes based on specific antipsychotic combinations rather than pooling data irrespective (111). The risks of adverse drug reactions due to psychotropic polypharmacy may be higher among certain regions in the world. Because of these aspects, the impact of psychotropic polypharmacy on COVID-19-related mortality therefore remains unknown.

Benzodiazepines (BZDs) and BZD-related medications (BZDRs) may also be of concern. A Swedish study in patients with SZ (112) showed that high exposure to BZDs is associated with an up to 70% higher mortality risk, compared with no users of BZDs. This is an important observation, knowing that the use of these medications by patients with SMI probably is even more common in other developed countries as the U.S (112). Although the risk of respiratory impairment associated with BZD use in the general population remains debated, in several studies current or recent exposure to certain BZDs or BZDRs has been found to be associated with an increased pneumonia risk (113), particularly in critically ill patients in intensive care units (114) or elderly (115, 116), whose immune system is vulnerable. BZDs and BZDRs, taken by 30–60% of individuals with SZ or BD (55), illnesses already characterized by a systemic pro-inflammatory state (see further), therefore may increase the risk for COVID-19-related mortality in these persons.

Finally, clinically relevant drug interactions between psychotropic medications and antiviral COVID-19 therapies may exist. The co-administration of protease inhibitors (blocking the protease enzyme that the virus needs to replicate), with certain antipsychotics (e.g., haloperidol and quetiapine), the mood stabilizer carbamazepine, or the BZDs midazolam or triazolam should be avoided because of increased toxicity and possible life-threatening events (117, 118).

Results of the studies included in our review indicate that COVID-19-related mortality risk, even after adjustment for all above mentioned factors, remains high in these patients (see Table 1). This indicates that SMI-related issues (i.c., immunological disturbances) may further increase the risk of COVID-19-related mortality. In one of the included studies [i.c., (43)] the high risk of mortality associated with schizophrenia spectrum disorders ranked second behind age in strength of an association among all known demographic and medical risk factors examined.

Research has shown that disease-related immune dysregulation may provide some explanation for the higher susceptibility of people with SMI for severe clinical outcomes of COVID-19 (42, 83, 119, 120). Hyperactivation of the immune system, leading to excess release of pro-inflammatory cytokines (hypercytokinemia) or a “cytokine storm” (cytokine release syndrome), seems to play a major role in the process of disease aggravation in patients with COVID-19 infection (121–127). Circulating levels of inflammatory biomarkers, including interleukin-1 (IL-1), interleukin-6 (IL-6), tumor necrosis factor alpha (TNF-α), and C-reactive protein (CRP), are often excessively elevated during severe SARS-CoV-2 infection. This disproportionate release of cytokines beyond that of a controlled immune response has been associated with poor outcomes and an increased risk of mortality (22, 122, 124, 126, 128, 129). As SMIs already are often characterized by a systemic pro-inflammatory state or overproduction of pro-inflammatory cytokines, which may persist even after patients' symptoms have improved (32, 33), the systemic hyperinflammation triggered by SARS-CoV-2 infection may be more pronounced in these individuals, leading to excess tissue damage, multi-organ failure, and death. A reduction in lymphocyte natural killer cell activity (a common finding in severe COVID-19) in some patients with SMI may further explain why COVID-19-related mortality rates are higher in these individuals (119, 120, 130–132). However, these hypotheses remain to be tested more rigorously.

Particularly in older patients, perturbations in gut microbiome composition, which seem to be related to elevated concentrations of inflammatory cytokines, may exacerbate COVID-19-related severity (133). This observation may be important knowing that people with SMI present with various alterations of the gut microbiome (134).

### Prevention Strategies and Possible Therapeutic Options

Given the strong association between COVID-19-related mortality and SMI, it is paramount that COVID-19 vaccination and equitable access to COVID-19 vaccines for people with SMI should be a matter of priority (135–137). This should be even more obvious knowing that the management of physical diseases (including comorbid conditions causing more severe COVID-19 illness) in people with SMI is already suboptimal, due to non-medical factors such as stigmatization and disparities in physical health care (10, 11, 13). It is therefore astonishing to note that some governments within and outside the European Union (e.g., India) are still doubting whether these individuals should be prioritized for COVID-19 vaccination (135).

However, only granting priority access to people with SMI in national vaccination strategies will not be sufficient, as a significant COVID-19 vaccination gap seems to exist between these individuals and the general population, despite having been granted early universal or priority access to SARS-CoV-2 vaccination (138–140). Targeted interventions to maximize vaccination uptake among these patients will be needed (139, 140). There are ways mental health professionals and agencies can address barriers to COVID-19 vaccination, based on the Increasing Vaccination Model (IVM). Identifying and addressing internal conflicts (by using motivational interviewing), social network interventions (making clinician recommendations build on interpersonal trust), and direct behavior change interventions (including reminders and primes, automatic appointments, and presumptive healthcare professional communication) can be helpful in this regard (141). Developing an intentional vaccine delivery strategy in conjunction with experts, utilizing multiple communication channels, and expanding vaccine delivery outside of the hospital to reach patients can be another strategy (142). Finally, the involvement of peers, family, or volunteers to support people with SMI in making healthcare choices may also be helpful. These types of actions can pay off. Our research group has shown that vaccine willingness among patients with psychiatric disorders in our university psychiatric hospital with a targeted prevention program was just as high as in the general population: 93% or 1,070 of 1,151 patients who were offered COVID-19 vaccination accepted this vaccination (143). Other studies confirmed that vaccination willingness among these patients is at least almost as high as in the general population (144, 145).

Several therapeutic options for this vulnerable population may exist to reduce the increased mortality rate: the use of medications that target specific inflammatory markers, the use of a cytokine filter targeting multiple different cytokines at a larger scale, natural killer cell-based immunotherapies, and the use of nicotine, nicotinic receptor agonists, or positive modulators of these receptors (activation of these receptors, particularly α7 nAChR, can suppress production of pro-inflammatory cytokines as these receptors are abundantly expressed in a variety of immune cells) (122, 127, 130, 131). Early prediction of a cytokine storm is made possible by several biochemical and hematological markers (128). In addition to reducing pathogen exposure, individual immunity in this vulnerable population can be enhanced by promoting a healthy lifestyle, regular exercise, balanced nutrition, and quality sleep (125). However, more research on these therapeutic options is urgently needed.

Patients with SMI often have lower vitamin D levels (146). Several meta-analyses (147, 148) and publications (146, 149–153) suggest that vitamin D supplementation may be potentially effective in preventing COVID-19 infection and mitigating the clinical course of the disease. Study results, however, remain difficult to interpret due to possible confounding factors (149, 154).

## Strengths and Limitations

Our review has particular strengths. Compared to other available systematic reviews, we developed a more comprehensive search strategy for the retrieval of reports of controlled trials. With the exception of Ceban et al. (38), our review is the only one that also included the Embase database to obtain a more comprehensive coverage of the existing US and EU literature. Finally, a thorough and critical discussion of this issue is presented in this paper.

Nevertheless, our systematic and critical review has its limitations that are inherent to the nature of the available evidence, and in that respect comparable to the previously published reviews. An important limitation is that all included studies are observational and mostly retrospective, and therefore causal relationships cannot be inferred. Most of the included studies in our systematic review were carried out during the first wave of the COVID-19 pandemic, which lasted from February/March until May/June 2020. During this period, in most countries testing was largely restricted to individuals exhibiting symptoms or to certain risk groups, due to a limited PCR-testing capacity at that time. This biased deployment of testing can distort true estimates of COVID-19-related mortality rate in people with SMI. Most of the studies on mood disorders involved mixed populations and did not make a distinction as to whether an individual with a mood disorder had MDD or BD. The two largest studies on COVID-19-related mortality risk in patients with BD to date (42, 155) only included around 500 patients. This is an important limitation given the possible differential impact of COVID-19-related mortality risk across SMIs. Therefore, more studies are needed that present separate data for patients with BD and MDD to gain a better estimate of true COVID-19-related mortality risks associated with these groups of people with SMI. Control groups also varied across studies: some control groups excluded patients with psychiatric disorders, while others included non-SMI disorders. Finally, most studies did not provide detailed patient sociodemographic characteristics (i.c., mean/median age, gender), or other detailed information on clinical/psychiatric variables (such as severity and status of the disease), and smoking histories. Future studies therefore should further explore these issues to better understand which specific patients are at an increased risk of COVID-19-related mortality.

## Conclusion

Even without taking COVID-19 into account, people with SMI already have a two to three times higher mortality rate than the general population, largely attributable to somatic comorbidities. Our review has shown that individuals with SMI, particularly patients with schizophrenia spectrum disorders, are at significantly higher risk of COVID-19-related mortality, not only due to higher somatic comorbidity rates and the possible use of psychotropic medication, but also to unknown factors at the moment that will have to be explored in future research. Severe mental illness therefore should be identified as a separate, independent risk factor for a more severe clinical course when infected with COVID-19 and targeted as a high-risk population. Consequently, targeted interventions to maximize vaccination uptake among these people should be prioritized in health policy worldwide.

## Data Availability Statement

The original contributions presented in the study are included in the article/Supplementary Material, further inquiries can be directed to the corresponding author/s.

## Author Contributions

JD: search and analysis. All authors contributed to the article and approved the submitted version.

## Conflict of Interest

The authors declare that the research was conducted in the absence of any commercial or financial relationships that could be construed as a potential conflict of interest.

## Publisher's Note

All claims expressed in this article are solely those of the authors and do not necessarily represent those of their affiliated organizations, or those of the publisher, the editors and the reviewers. Any product that may be evaluated in this article, or claim that may be made by its manufacturer, is not guaranteed or endorsed by the publisher.
