# Supplementary material for: COVID-19-Related Mortality Risk in People With Severe Mental Illness: A Systematic and Critical Review
Source: Front Psychiatry. 2022 Jan 13;12:798554. doi: 10.3389/fpsyt.2021.798554 (PMC8793909; doi:10.3389/fpsyt.2021.798554)
Supplement: Supplementary file 1 [file Data_Sheet_1.docx]

**SUPPLEMENT S1**

**SEARCH STRINGS**

**PUBMED**

("Coronavirus"[Mesh:NoExp] OR "Betacoronavirus"[Mesh:NoExp] OR "Coronavirus Infections"[Mesh:NoExp] OR "COVID-19"[MeSH] OR "coronavirinae"[tiab] OR nCoV[tiab] OR 2019nCoV[tiab] OR COVID[tiab] OR COVID19[tiab] OR "severe acute respiratory syndrome 2"[tiab] OR nCoV[ad] OR 2019nCoV[ad] OR COVID[ad] OR COVID19[ad] OR "SARS-Cov-2"[MeSH] OR "severe acute respiratory syndrome 2"[ad] OR SARS2[ad] OR "cov 2"[ad] OR cov2[ad] OR coronavirus*[ad] OR betacoronavirus*[ad] OR "corona virus*"[ad] OR SARS2[tiab] OR "cov 2"[tiab] OR cov2[tiab] OR coronavirus*[tiab] OR betacoronavirus*[tiab] OR "corona virus*"[tiab] OR "Wuhan virus"[tiab] OR ((wuhan[tiab] OR novel[tiab] OR new[tiab] OR 19[tiab] OR 2019[tiab] OR epidem*[tiab] OR pandem*[tiab] OR outbreak[tiab] OR wuhan[ad] OR novel[ad] OR new[ad] OR 19[ad] OR 2019[ad] OR epidemy[ad] OR epidemic*[ad] OR pandem*[ad] OR outbreak[ad]) AND ("pneumonia virus*"[tiab] OR cov[tiab] OR hcov[tiab]))) AND ("Schizophrenia Spectrum and Other Psychotic Disorders"[Mesh] OR "Bipolar and Related Disorders"[Mesh] OR "Mood Disorders"[Mesh] OR "severe mental illness*"[tiab] OR "severe mental disorder*"[tiab] OR "major psychiatric disorder*"[tiab] OR "major psychiatric illness*"[tiab] OR "schizophreni*"[tiab] OR "schizoaffective*"[tiab] OR "schizo-affective*"[tiab] OR "delusional disorder*"[tiab] OR "paranoid disorder*"[tiab] OR "schizotypal disorder*"[tiab] OR "psychotic disorder*"[tiab] OR "psychosis"[tiab] OR "catatoni*"[tiab] OR "bipolar and related disorder*"[tiab] OR "bipolar disorder*"[tiab] OR "bipolar I disorder*"[tiab] OR "bipolar type I disorder*"[tiab] OR "bipolar II disorder*"[tiab] OR "bipolar type II disorder*"[tiab] OR "cyclothymi*"[tiab] OR "manic depressi*"[tiab] OR "manic episode*"[tiab] OR "mania"[tiab] OR "affective disorder*"[tiab] OR "bipolar illness"[tiab] OR "mood disorder*"[tiab] OR "depressive disorder*"[tiab] OR "major depression"[tiab] OR "MDD"[tiab] OR "agitated depression"[tiab] OR "reactive depression"[tiab] OR "vital depression"[tiab] OR "disruptive mood dysregulation disorder*"[tiab] OR "dysthymi*"[tiab] OR "disthymi*"[tiab] OR "dysthimi*"[tiab] OR "premenstrual dysphoric disorder*"[tiab]) AND ("Mortality"[Mesh] OR "Death"[Mesh:NoExp] OR "Odds Ratio"[Mesh] OR "mortalit*"[tiab] OR "death*"[tiab] OR "fatal*"[tiab] OR "lethal*"[tiab] OR "SMR*"[tiab] OR "risk ratio*"[tiab] OR "odds ratio*"[tiab] OR "hazard ratio*"[tiab])

**EMBASE**

('Coronavirinae'/de OR 'Betacoronavirus'/de OR 'Coronavirus infection'/de OR 'coronavirus disease 2019'/exp OR 'nCoV':ti,ab,kw,ff OR '2019nCoV':ti,ab,kw,ff OR 'COVID':ti,ab,kw,ff OR 'COVID19':ti,ab,kw,ff OR 'Severe acute respiratory syndrome coronavirus 2'/exp OR 'severe acute respiratory syndrome 2':ti,ab,kw,ff OR 'SARS2':ti,ab,kw,ff OR 'cov 2':ti,ab,kw,ff OR 'cov2':ti,ab,kw,ff OR 'coronavirus*':ti,ab,kw,ff OR 'betacoronavirus*':ti,ab,kw,ff OR 'coronavirinae':ti,ab,kw OR 'corona virus*':ti,ab,kw,ff OR 'Wuhan virus':ti,ab,kw OR (('wuhan':ti,ab,kw,ff OR 'novel':ti,ab,kw,ff OR 'new':ti,ab,kw,ff OR '19':ti,ab,kw,ff OR '2019':ti,ab,kw,ff OR 'epidem*':ti,ab,kw OR 'pandem*':ti,ab,kw,ff OR 'outbreak':ti,ab,kw,ff OR 'epidemy':ff OR 'epidemic*':ff) AND ('pneumonia virus*':ti,ab,kw OR 'cov':ti,ab,kw OR 'hcov':ti,ab,kw))) AND ('psychosis'/exp OR 'schizophrenia spectrum disorder'/exp OR 'mood disorder'/exp OR 'disruptive mood dysregulation disorder'/exp OR 'severe mental illness'/exp OR 'severe mental disorder'/exp OR 'severe mental illness*':ti,ab,kw OR 'severe mental disorder*':ti,ab,kw OR 'major psychiatric disorder*':ti,ab,kw OR 'major psychiatric illness*':ti,ab,kw OR 'psychosis':ti,ab,kw OR 'schizophreni*':ti,ab,kw OR 'schizoaffective*':ti,ab,kw OR 'schizo-affective*':ti,ab,kw OR 'delusional disorder*':ti,ab,kw OR 'paranoid disorder*':ti,ab,kw OR 'schizotypal disorder*':ti,ab,kw OR 'psychotic disorder*':ti,ab,kw OR 'catatoni*':ti,ab,kw OR 'mania':ti,ab,kw OR 'bipolar disorder*':ti,ab,kw OR 'bipolar and related disorder*':ti,ab,kw OR 'bipolar I disorder*':ti,ab,kw OR 'bipolar type I disorder*':ti,ab,kw OR 'bipolar II disorder*':ti,ab,kw OR 'bipolar type II disorder*':ti,ab,kw OR 'cyclothymi*':ti,ab,kw OR 'manic depressi*':ti,ab,kw OR 'manic episode*':ti,ab,kw OR 'affective disorder*':ti,ab,kw OR 'bipolar illness':ti,ab,kw OR 'mood disorder*':ti,ab,kw OR 'depressive disorder*':ti,ab,kw OR 'major depression':ti,ab,kw OR 'MDD':ti,ab,kw OR 'agitated depression':ti,ab,kw OR 'reactive depression':ti,ab,kw OR 'vital depression':ti,ab,kw OR 'disruptive mood dysregulation disorder*':ti,ab,kw OR 'dysthymi*':ti,ab,kw OR 'disthymi*':ti,ab,kw OR 'dysthimi*':ti,ab,kw OR 'premenstrual dysphoric disorder*':ti,ab,kw) AND ('mortality'/de OR 'mortalit*':ti,ab,kw OR 'hospital mortality'/exp OR 'mortality rate'/exp OR 'premature mortality'/exp OR 'standardized mortality ratio'/exp OR 'odds ratio'/exp OR 'hazard ratio'/exp OR 'risk ratio'/exp OR 'death*':ti,ab,kw OR 'fatal*':ti,ab,kw OR 'SMR*':ti,ab,kw OR 'risk ratio*':ti,ab,kw OR 'hazard ratio*':ti,ab,kw OR 'odds ratio*':ti,ab,kw OR 'death'/de OR 'fatality'/exp OR 'lethality'/exp OR 'lethal*':ti,ab,kw)

**WEB OF SCIENCE**

(TS=("coronavirus*" OR "betacoronavirus*" OR "COVID-19" OR "coronavirinae" OR "nCoV" OR "2019nCoV" OR "COVID" OR "COVID19" OR "severe acute respiratory syndrome 2" OR "SARS2" OR "cov 2" OR "cov2" OR "corona virus*" OR "Wuhan virus" OR (("wuhan" OR "novel" OR "new" OR "19" OR "2019" OR epidem* OR pandem* OR "outbreak") AND ("pneumonia virus*" OR "cov" OR "hcov"))) OR AD=("nCoV" OR "2019nCoV" OR "COVID" OR "COVID19" OR "SARS2" OR "cov 2" OR "cov2" OR "coronavirus*" OR "betacoronavirus*" OR "corona virus*" OR "severe acute respiratory syndrome 2") OR (AD=("wuhan" OR "novel" OR "new" OR "19" OR "2019" OR "epidemy" OR "epidemic*" OR "pandem*" OR "outbreak") AND TS=("pneumonia virus*" OR "cov" OR "hcov"))) AND TS=("severe mental illness*" OR "severe mental disorder*" OR "major psychiatric illness*" OR "major psychiatric disorder*" OR schizophreni* OR schizoaffective* OR "schizo-affective*" OR "delusional disorder*" OR "paranoid disorder*" OR "schizotypal disorder*" OR "psychotic disorder*" OR "psychosis" OR catatoni* OR "bipolar and related disorder*" OR "bipolar disorder*" OR "bipolar I disorder*" OR "bipolar type I disorder*" OR "bipolar II disorder*" OR "bipolar type II disorder*" OR cyclothymi* OR "manic depressi*" OR "manic episode*" OR mania OR "affective disorder*" OR "bipolar illness" OR "mood disorder*" OR "depressive disorder*" OR "major depression" OR "MDD" OR "agitated depression" OR "reactive depression" OR "vital depression" OR "disruptive mood dysregulation disorder*" OR dysthymi* OR disthymi* OR dysthimi* OR "premenstrual dysphoric disorder*") AND TS=("mortalit*" OR "death*" OR "fatal*" OR "lethal*" OR "SMR*" OR "risk ratio*" OR "odds ratio*" OR "hazard ratio*")

**SCOPUS**

(TITLE-ABS(coronavirus* OR betacoronavirus* OR coronavirinae OR nCoV OR 2019nCoV OR COVID OR COVID19 OR "severe acute respiratory syndrome 2" OR SARS2 OR "cov 2" OR cov2 OR "corona virus*" OR "Wuhan virus") OR AFFIL(nCoV OR 2019nCoV OR COVID OR COVID19 OR “severe acute respiratory syndrome 2” OR SARS2 OR "cov 2" OR cov2 OR "coronavirus*" OR betacoronavirus* OR "corona virus*") OR ((TITLE-ABS(wuhan OR novel OR new OR 19 OR 2019 OR epidem* OR pandem* OR outbreak) OR AFFIL(wuhan OR novel OR new OR 19 OR 2019 OR epidemy OR epidemic* OR pandem* OR outbreak)) AND TITLE-ABS("pneumonia virus*" OR cov OR hcov))) AND (TITLE-ABS(schizophreni* OR schizoaffective* OR schizo-affective* OR "delusional disorder*" OR "paranoid disorder*" OR "schizotypal disorder*" OR "psychotic disorder*" OR psychosis OR catatoni* OR "bipolar and related disorder*" OR "bipolar disorder*" OR "bipolar I disorder*" OR "bipolar type I disorder*" OR "bipolar II disorder*" OR "bipolar type II disorder*" OR cyclothymi* OR "manic depressi*" OR "manic episode*" OR mania OR "affective disorder*" OR "bipolar illness" OR "mood disorder*" OR "depressive disorder*" OR "major depression" OR MDD OR "agitated depression" OR "reactive depression" OR "vital depression" OR "disruptive mood dysregulation disorder*" OR dysthymi* OR disthymi* OR dysthimi* OR "premenstrual dysphoric disorder*") AND TITLE-ABS("mortalit*" OR "death*" OR "fatal*" OR "lethal*" OR "SMR*" OR "risk ratio*" OR "odds ratio*" OR "hazard ratio*"))

**SUPPLEMENT S2**

**Table 2A: Quality assessment of the included cohort studies using the Newcastle-Ottawa Scale (NOS)**

| Study | Representativeness of the exposed cohort | Selection of the non-exposed cohort | Ascertainment of exposure | Comparability of cohorts on the basis of the design or analysis | Outcome assessment | Psychiatric diagnoses occurred prior to COVID-19 diagnoses | Total | Evaluation |
| --- | --- | --- | --- | --- | --- | --- | --- | --- |
| Barcella et al. (2021) | * | * | * | * | * | ***** | 6/7 | High |
| Nemani et al., (2021) | * | * | * | ** | * | * | 7/7 | High |
| Tzur Bitan et al. (2021) | * | * | * | * | * | * | 6/7 | High |
| Jeon et al. (2021) | * | * | * | * | * | * | 6/7 | High |
| Fond et al. (2021b) | * | * | * | ** | * | * | 7/7 | High |
| Reilev et al. (2020) | * | * | * | * | * | * | 6/7 | High |
| Poblador-Plou et al. (2020) | * | * | * | * | * | * | 6/7 | High |
| Yang et al. (2020) | * | * | * | ** | * | * | 7/7 | High |
| Castro et al. (2021) |  | * | * | * | * | * | 5/7 | Moderate |
| Diez-Quevedo et al. (2021) |  | * | * | * | * | ***** | 5/7 | Moderate |

**Table 2B: Quality assessment of the included case-control studies using the Newcastle-Ottawa Scale (NOS)**

| Study | Adequate case definition | Representativeness of the cases | Selection of controls | Definition of controls | Comparability of cases and controls on the basis of the design or analysis | Ascertainment of exposure | Same method of ascertainment for cases and controls | SMI diagnoses prior to COVID-19 diagnoses | Total | Evaluation |
| --- | --- | --- | --- | --- | --- | --- | --- | --- | --- | --- |
| Fond et al. (2021d) | * | * | * | * | ** | * | * | * | 9/9 | High |
| Wang et al. (2021) |  | * | * |  | * | * | ***** | * | 6/9 | Moderate |

**Table 2C: Quality assessment of the included cross-sectional studies using the Newcastle-Ottawa Scale (NOS)***

| Study | Representativeness of the sample | Sample size justified and satisfactory | Adequate case d efinition | Comparability of subjects in different outcome groups | Ascertainment of exposure | Appropriate statistical testing | Total | Evaluation |
| --- | --- | --- | --- | --- | --- | --- | --- | --- |
| Egede et al. (2021) | * |  | * | * | * | * | 5/7 | Moderate |

*The NOS was adapted for cross-sectional data
